# Identifying Malnutrition in an Elderly Ambulatory Rehabilitation Population: Agreement between Mini Nutritional Assessment and Validated Screening Tools

**DOI:** 10.3390/healthcare3030822

**Published:** 2015-09-11

**Authors:** Alison Yaxley, Maria Crotty, Michelle Miller

**Affiliations:** 1 Nutrition and Dietetics, School of Health Sciences, Faculty of Medicine, Nursing and Health Sciences, Flinders University, GPO Box 2100, Adelaide, SA 5001, Australia; E-Mail: michelle.miller@flinders.edu.au; 2 Rehabilitation, Aged and Extended Care, School of Health Sciences, Faculty of Medicine, Nursing and Health Sciences, Flinders University, GPO Box 2100, Adelaide, SA 5001, Australia; E-Mail: maria.crotty@flinders.edu.au

**Keywords:** malnutrition, older adult, screening, rehabilitation

## Abstract

Malnutrition is common in older adults and often goes unrecognised and untreated. Australian evidence-based guidelines for the management of malnutrition indicate that only the Mini Nutritional Assessment short form (MNA-sf) and Rapid Screen are recommended for use as malnutrition screening tools in the rehabilitation setting. The aim of this secondary analysis was to assess the validity and reliability of two malnutrition screening tools, validated in other adult sub-groups, in a rehabilitation population aged ≥60 years. The Council on Nutrition Appetite Questionnaire (CNAQ) and the Simplified Nutritional Appetite Questionnaire (SNAQ), were completed by 185 ambulatory rehabilitation patients (48% male; median age 78 years) and results compared to the full MNA as a reference technique. Prevalence of risk of malnutrition was 63% according to the MNA. For identification of risk of malnutrition the CNAQ had sensitivity of 54%, specificity 81%, positive predictive value 83% and negative predictive value 51%, compared to 28%, 94%, 89% and 44%, respectively, using SNAQ. Assessment of reliability indicated significant slight to fair agreement between MNA with CNAQ (κ = 0.309, *p* < 0.001) and SNAQ (κ = 0.176, *p* < 0.001). Neither the CNAQ nor the SNAQ have a high level of validity or reliability in this elderly population and are therefore not recommended for use in the ambulatory rehabilitation setting. Further work is necessary to assess the validity and reliability of other malnutrition screening tools to establish their usefulness in this population.

## 1. Introduction

Malnutrition, used here to refer to under nutrition, is widespread in older adults across all settings [1]. It is well documented that malnutrition is common amongst the ambulatory rehabilitation population, particularly older adults, and often goes unrecognised. The prevalence in rehabilitation has been reported as 30%–50% [1], however recent data indicate that 84% of older adults in ambulatory rehabilitation in two rehabilitation hospitals in New South Wales were malnourished or at risk of malnutrition [2]. It is not likely that these numbers will improve with forecasts that those aged 65 years or more will comprise around 22% of the Australian population by 2061 [3].

Malnutrition is costly to individuals and the hospital system alike, in terms of negative health impacts such as increased length of stay, delayed wound healing, and increased risk of infection [4]. In addition, the monetary costs to the health system are staggering. The cost of malnutrition to the hospital system is estimated to be approximately $10.7 million annually in one Australian state alone [5] and the cost of hospital treatment is 20% higher for each nutritionally at risk patient [6]. As malnutrition is often undiagnosed and therefore unreported hospitals may be missing out on reimbursements which may indicate that these costs are significantly underestimated. Furthermore, those at risk of malnutrition may find it difficult to meet the increased physical and mental demands of a rehabilitation program, hindering their chances of success [2].

Effective prevention and treatment of malnutrition relies on accurate diagnosis. Nutrition screening identifies individuals who are at risk of becoming malnourished. There is good evidence to support routine nutrition screening in all settings using a valid tool which is appropriate to the population in which it is to be applied [1]. Numerous malnutrition screening tools are used in practice, however the Dietitians Association of Australia (DAA) endorsed Evidence Based Practice Guidelines for the Nutritional Management of Malnutrition in Adult Patients Across the Continuum of Care recommend only the Mini Nutritional Assessment-Short Form (MNA-SF) and the Rapid Screen for use in ambulatory rehabilitation [1]. Other tools have not yet been found to be valid and reliable in this group.

As indicated previously there are many other validated malnutrition screening tools in existence and many have been shown to effectively identify the risk of malnutrition in a variety of other population groups. Two of those tools, the Council on Nutrition Appetite Questionnaire (CNAQ) and the Simplified Nutritional Appetite Questionnaire (SNAQ), which were developed in community dwelling adults and long term care residents [7], have been found to be valid and reliable in community, acute and aged care settings but have not been assessed for use in the rehabilitation population. While the MNA-SF and the Rapid Screen contain objective measures, and may therefore be more accurate, this may limit their usefulness in practice as some level of skill is required to perform accurate measures, which can also be time consuming. The subjective self-report questions included in the CNAQ and SNAQ may therefore make them more attractive for use in this setting.

Thus, this study aimed to test the validity and reliability of the SNAQ and the CNAQ to effectively detect risk of malnutrition in a sample of older adults participating in ambulatory rehabilitation.

## 2. Experimental Section

### 2.1. Participants and Setting

A secondary analysis was performed on baseline data collected as part of a randomized controlled trial (RCT) conducted at three public hospitals in southern Adelaide, Australia between June 2005 and June 2006. The RCT compared the effects of a hospital-based day rehabilitation program and a home-based rehabilitation program, and the methods used in the trial were previously described in detail [8]. All participants had been referred for ambulatory rehabilitation following an acute hospital admission. Patients were eligible if they were medically stable, ready for discharge from hospital and had rehabilitation goals that necessitated at least 12 therapy sessions. Those aged ≥60 years were included in this secondary analysis. The RCT was conducted in accordance with the Declaration of Helsinki, and the protocol was approved by the Clinical Research Ethics Committees at Repatriation General Hospital, Flinders Medical Centre and Noarlunga Health Services in Adelaide, Australia. All study participants gave written informed consent to inclusion before they participated in the study. Data to describe the sample were collected from medical records at baseline.

### 2.2. Nutritional Status

**Figure 1:**
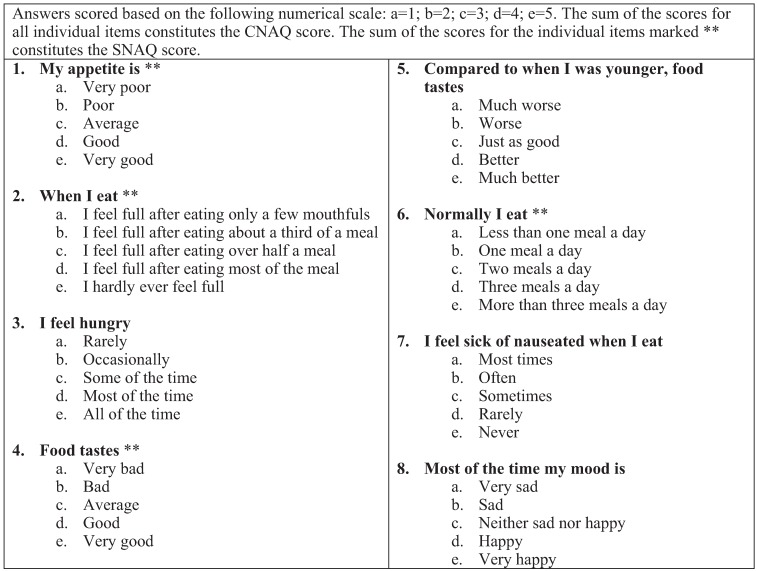
The Council on Nutrition Appetite Questionnaire (CNAQ) and the Simplified Nutritional Appetite Questionnaire (SNAQ) tools used in the randomised controlled trial of elderly ambulatory rehabilitation patients [7].

Study participants completed two self-reported malnutrition screening tools; the CNAQ and the SNAQ (Figure 1) [7]. The CNAQ is an 8-item questionnaire which has been found to be valid in predicting clinically significant weight loss in older adults in community dwelling older adults and in long term care residents [7]. Each item is scored on a 5-point scale with possible total score ranging from 8 (worst) to 40 (best). A score of ≤28 indicates significant risk of at least 5% weight loss within 6 months and such participants were classified as at risk of malnutrition. The SNAQ is a 4-item derivative of the CNAQ found to be similarly valid [7]. With a similar scoring rubric, but fewer items possible, total score for the SNAQ ranges from 4 (worst) to 20 (best). Scores ≤14 indicate significant risk of at least 5% weight loss within 6 months and such participants were classified as at risk of malnutrition. All other participants were classified as well-nourished.

The nutritional status of study participants was assessed by a trained research dietitian using the Mini Nutritional Assessment (MNA) [9], an 18-item assessment tool developed for use with older adults and validated in a number of different settings including rehabilitation [10]. Participants who were identified as at risk of malnutrition (score 17–23.5) or malnourished (score < 17) were grouped together and categorised as at risk of malnutrition. This single group of participants who scored ≤23.5 was created in order to reflect the results of the malnutrition screening tools which identify those at risk of malnutrition and cannot recognize malnutrition *per se*. All other participants were classified as well-nourished (score 24–30).

### 2.3. Statistical Analysis

Data collected as part of this study were analysed using SPSS version 17.0 [11] and the level of statistical significance was set at *p* < 0.05. After checking the distribution for normality sample characteristics were described by median (interquartile range (IQR)) for continuous variables and frequencies (%) for categorical variables. Contingency tables were used to determine sensitivity, specificity, positive predictive values (PPV) and negative predictive values (NPV) of both the CNAQ and the SNAQ, in comparison to the MNA. Kappa measure of agreement was used to assess consistency between the CNAQ and SNAQ, and the MNA and results categorised according to the classification of Landis and Koch [12].

## 3. Results and Discussion

### 3.1. Results

From 225 ambulatory rehabilitation participants who were randomized in the primary RCT, 185 were ≥60 years and had data available for inclusion in this secondary analysis. The sample was 48% male and had median (IQR) age of 78 (71.5, 83.0) years. According to the MNA 63% of study participants were at risk of malnutrition. Sensitivity and specificity and PPV and NPV of CNAQ and SNAQ, compared to MNA, were calculated from the contingency tables displayed in Table 1 and Table 2.

**Table 1:** Contingency table presenting classification of nutritional status of ambulatory rehabilitation participants by CNAQ and Mini Nutritional Assessment (MNA).

		MNA	
		At Risk	Well Nourished
CNAQ	At risk	62	13
	Well nourished	54	56

**Table 2:** Contingency table presenting classification of nutritional status of ambulatory rehabilitation participants by SNAQ and MNA.

		MNA	
		At Risk	Well Nourished
SNAQ	At risk	32	4
	Well nourished	84	65

When compared to the MNA, the CNAQ demonstrated sensitivity of 54%, specificity of 81%, PPV of 83% and NPV of 51%, compared to 28%, 94%, 89% and 44%, respectively, using the SNAQ.

Assessment of reliability between the MNA and CNAQ and SNAQ resulted in statistically significant slight agreements with κ = 0.309 (*p* < 0.001) and κ = 0.176 (*p* < 0.001), respectively.

### 3.2. Discussion

According to the MNA 63% of participants in this study were at risk of malnutrition. Results from this secondary analysis of 185 ambulatory rehabilitation patients indicate that, in comparison to the MNA, neither the CNAQ nor the SNAQ have a high level of validity or reliability for the identification of risk of malnutrition in this group of frail older adults.

The literature indicates that nutrition screening tools demonstrating levels of sensitivity, specificity, PPV and NPV of ≥70% [13] reflect adequate levels of validity. Furthermore, levels of ≤60% are reported to be poor. While an ideal screening tool would have sensitivity and specificity of 100%, in practice there would more usually be a tradeoff such that high sensitivity results in low specificity and vice versa. While both the CNAQ and the SNAQ demonstrate specificity and PPV at acceptable levels, the levels of sensitivity and NPV are poor. Low sensitivity for both screening tools demonstrates that they are inaccurate in detecting risk of malnutrition in this group of older adults. Results indicate that up to 46% of those screened using the CNAQ and up to 72% of those screened using the SNAQ would be classified as well-nourished when in fact they were at risk of malnutrition. The consequence of this lack of recognition is a lack of appropriate intervention with subsequent deterioration in nutritional status and poor outcomes for the patient. These findings are in contrast to sensitivity and specificity in excess of 80% for CNAQ, and in excess of 75% for the SNAQ, reported in community dwelling adults and long term care residents [7]. The literature indicates that the nutritional status of rehabilitation patients is poor [1,2] however selection of tools with more acceptable levels of validity, particularly high sensitivity, would likely minimize the deterioration of nutritional status in this group by highlighting those at risk and enabling appropriate and timely intervention.

Sensitivity and specificity are an indication of those who are truly positive, or truly negative, for a test, in this case who are or are not at risk of malnutrition. The predictive values however provide an assessment of the diagnostic accuracy of a test. The low levels of NPV for CNAQ (51%) and SNAQ (44%) indicate that, among those who were identified as well-nourished, approximately half were in fact at risk of malnutrition, with similar consequences to the low levels of sensitivity already discussed.

A recent study conducted in Malaysia attempted to validate the CNAQ and the SNAQ against the Subjective Global Assessment in hospitalized older adults and outpatients [14]. The results indicate better sensitivity and PPV than the current study, although lower specificity, and NPV around the same level, with moderate reliability for both tools. Reasons for these differences are not clear however possibilities include differences in body type between Malaysian and Caucasian older adults which may influence results of the SGA, and the difference in clinical setting between the current study and that carried out in Malaysia which may render any comparison between the two impractical.

Reliability of both tools in comparison to the MNA, although statistically significant, was below acceptable levels. While the CNAQ demonstrated fair agreement (κ = 0.309) the SNAQ showed only slight agreement (κ = 0.176) [12]. These levels of agreement are very low when compared with other malnutrition screening tools used in a range of settings in Australia, such as the Malnutrition Screening Tool (κ = 0.84–0.93) [15] and the Malnutrition Universal Screening Tool (κ = 0.8–1.0) [16]. However, neither of these tools have been validated for use in rehabilitation patients. The tools which are recommended for use in this setting by the DAA endorsed Evidence Based Practice Guidelines for the Nutritional Management of Malnutrition in Adult Patients Across the Continuum of Care are the MNA-SF (κ = 0.63) [17] and the Rapid Screen, which does not appear to have been tested for reliability.

The characteristics of a good malnutrition screening tool are that it is quick, simple and easy to use [18]. Both the CNAQ and the SNAQ possess those characteristics as they require no complex measurements and can be self-completed. However, the SNAQ is derived from the CNAQ and therefore may not be the obvious choice to be validated alongside the longer version. If the SNAQ had been more valid and reliable than its parent tool practitioners would have been able to offer a less burdensome screening tool to ambulatory rehabilitation patients. This was not the case and as has been indicated previously neither tool is an appropriate choice for this setting. This is not reflective of the literature; the paper reporting on development of the SNAQ indicates that the tool was developed to remove the “reliability reducing” items from the CNAQ [7]. The CNAQ and SNAQ were developed in community dwelling older adults and long term care residents which may account for this difference as the participants in the current study had recently been acutely unwell thus their answers may reflect that period.

There are some strengths and limitations of the current study that should be acknowledged. The participants in the original RCT ranged in age from 18 years to 94 years however only those aged ≥60 years were included in this secondary analysis. Of those in the primary study, 13 (6%) were aged less than 50 years, and 82% of participants were aged 60 years or over with a median age of 76 years. This reflects the patient profile reported recently in South Australia [19], and New South Wales [20], which indicates that the sample was likely representative of the wider ambulatory rehabilitation population, and thus that the findings using the whole group would be generalisable. However, the nutrition assessment tool used in the study, the MNA, was developed and validated for use in older adult populations hence it may not have been appropriate for a sample with such a wide age range. Nonetheless, a major strength of this study is the sample size. The literature indicates that validation studies should recruit 100 to 200 participants with over 200 participants adding little precision to findings [21]. The sample size of 185 participants in this study is thus likely appropriate to allow the authors to be confident in the findings.

## 4. Conclusions

Based on the results of this secondary analysis, the CNAQ and SNAQ do not have a high level of validity or reliability for the identification of malnutrition risk and are therefore not recommended for use in older ambulatory rehabilitation patients. There is however scope in the future to determine validity and reliability of other existing malnutrition screening tools to establish their usefulness in this population.
